# Preparation and Characterization of Oxidized Inulin Hydrogel for Controlled Drug Delivery

**DOI:** 10.3390/pharmaceutics11070356

**Published:** 2019-07-22

**Authors:** Franklin Afinjuomo, Paris Fouladian, Ankit Parikh, Thomas G. Barclay, Yunmei Song, Sanjay Garg

**Affiliations:** School of Pharmacy and Medical Sciences, University of South Australia, Adelaide, South Australia 5001, Australia

**Keywords:** hydrogels, oxidized inulin, periodate oxidation, colon targeting

## Abstract

Inulin-based hydrogels are useful carriers for the delivery of drugs in the colon-targeted system and in other biomedical applications. In this project, inulin hydrogels were fabricated by crosslinking oxidized inulin with adipic acid dihydrazide (AAD) without the use of a catalyst or initiator. The physicochemical properties of the obtained hydrogels were further characterized using different techniques, such as swelling experiments, in vitro drug release, degradation, and biocompatibility tests. The crosslinking was confirmed with Fourier transform infrared spectroscopy (FTIR), thermal gravimetric analysis (TGA), and differential scanning calorimetry (DSC). In vitro releases of 5-fluorouracil (5FU) from the various inulin hydrogels was enhanced in acidic conditions (pH 5) compared with physiological pH (pH 7.4). In addition, blank gels did not show any appreciable cytotoxicity, whereas 5FU-loaded hydrogels demonstrated efficacy against HCT116 colon cancer cells, which further confirms the potential use of these delivery platforms for direct targeting of 5-FU to the colon.

## 1. Introduction

Inulin is an important fructan found in plants and vegetables, such as garlic, leeks, bananas, and Jerusalem artichokes [1]. Inulin belongs to the fructan class of polysaccharides typically containing about 2–60 fructose units in linear chains with β(2→1) glycosidic linkages and typically linked to a terminal glucose unit [2]. There is a growing interest in inulin because of the key role that fructans play in both food and the pharmaceutical industry. Inulin is only digested by colon bacteria and not in the small intestine, promoting its health benefits as a prebiotic and in food [3,4,5]. Inulin also serves as a replacement for fat and sugar in the food industry [6,7]. Inulin is Food and Drug Administration (FDA) approved Generally Recognized As Safe (GRAS) polysaccharide with excellent biodegradability, biocompatibility, water solubility, renewability, and non-toxicity, making it very attractive as a drug delivery carrier targeting colon delivery and pulmonary delivery [8,9,10,11,12,13]. Other important applications include use as a flocculant for wastewater treatment [14] and in tissue engineering scaffolds [15]. The modification of inulin hydroxyl groups allows the introduction of new functional groups into the polymer. In addition to the multiple hydroxyl groups, the flexible furanose backbone, as well as its increased solubility compared with other polysaccharides, means that it can be readily modified chemically. This allows the use of inulin derivatives as carriers for a variety of pharmaceutical applications. Inulin is considered to be a very attractive polysaccharide for colon targeting, because it is only degraded by specific inulinase enzymes found in the colon [9].

For this reason, we hope to exploit the variation in the microflora concentration of various segments of the gastrointestinal tract for site-specific delivery of drugs to the colon. The chemical modification or derivatization of hydrophilic inulin results in new materials with hydrophobic characters making them suitable for drug delivery systems (DDSs), including hydrogels [16,17,18,19], micelles [20,21,22], liposomes [23], nanoparticles [24,25,26], vaccine adjuvants [27,28,29], solid dispersion [30,31], microparticles [8,12,32], and macromolecular bioconjugates/prodrugs [33,34]. To develop hydrogel drug delivery systems, different chemical strategies and techniques have been utilized for hydrogel synthesis. Reported methods of preparation include radical polymerization of inulin derivatives [10,11], Michael-addition crosslinking [35], condensation reaction [36], chemical crosslinking using crosslinkers [5,16,37], or chemical crosslinking followed by UV radiation [19] with subsequent formation of pH-sensitive inulin hydrogels. The limitations with the methods reported above include the use of a toxic catalyst [10,16], initiators [36,38], several reaction steps during synthesis, and long gelation period [37]. The use of modified polysaccharides with aldehyde functionality crosslinked with adipic acid hydrazide has been reported for biodegradable polysaccharides, such as pectin [39,40], dextran [41], chitosan [42], alginate [43,44], xanthan, hyaluronic acid [45,46,47,48], and gum with potential application as injectable hydrogel. Periodate oxidation of inulin is a well-known method of functionalizing inulin with an aldehyde group [33,49]. This enables the use of oxidized inulin as a macromolecular bioconjugate to couple a cardiac depressant and antiarrhythmic agent procainamide [33], as well as an enzyme immobilizing agent [49].

**Scheme 1:**
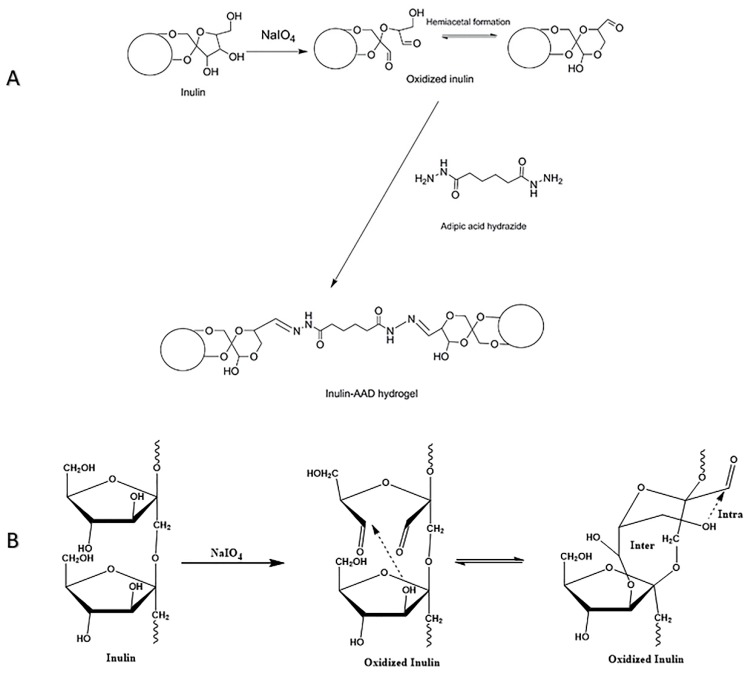
(**A**) Periodate oxidation of inulin and subsequent crosslinking with adipic acid dihydrazide (AAD) and (**B**) periodate oxidation of inulin with cleavage at C_3_–C_4_.

However, the use of oxidized inulin for hydrogel fabrication by crosslinking the polyaldehyde inulin with adipic acid hydrazide (AAD) has yet to be reported. To the best of our knowledge, there are no prior studies on the use of oxidized inulin in the formation of a hydrogel via crosslinking with non-toxic AAD. In this work, sodium periodate was used as an oxidizing agent to attack the hydroxyl group of inulin between C_3_–C_4_, breaking the C–C bond and producing two aldehyde groups (Scheme 1A,B). The resulting intermediate obtained from periodate oxidation is highly reactive toward nucleophilic compounds, such as carbazates, amines, and hydrazines. This oxidized inulin reacts with AAD to form a crosslink without the use of a catalyst. It is worth mentioning that this inulin hydrogel was fabricated in physiologic pH conditions (pH 7.4) in phosphate-buffered saline (PBS) as compared with the organic solvents used in most reported inulin hydrogels. Other clear advantages include (1) the fact that the oxidized inulin/AAD hydrogels were formed within 2–4 min and (2) the potential to control their mechanical and degradation properties with the amount of crosslinker used.

We hypothesized that the chemical crosslinking of oxidized inulin with different ratios of AAD would result in hydrogels with different degrees of crosslinking density, which can allow the controlled release of 5-fluorouracil (5FU). The aim of this study was to crosslink oxidized inulin with AAD to obtain a hydrogel with a hydrazone bond. The oxidized inulin was characterized using ^1^H NMR and FTIR, as well as colorimetric titration. The three hydrogels formed were evaluated using different techniques, such as swelling experiments, rheological measurement, FTIR, TGA, differential scanning calorimetry (DSC), SEM, and degradation as well as biocompatibility and cytotoxicity studies using 33-(4,5-dimethylthiazol-2-yl)-2,5-diphenyl-tetrazolium bromide blue-indicator dye (MTT) assay.

## 2. Materials and Methods

### 2.1. Materials

Inulin polysaccharide from a Dahlia plant was purchased from Sigma-Aldrich (Castle Hill, New South Wales, Australia), and the average chain length (DPn) 39.2 and MWn = 6381.7 was determined by using end-group analysis via an 1H NMR spectroscopy method previously reported by Barclay et al. [50]. Adipic acid dihydrazide, phosphate-buffered saline tablets, 5-fluorouracil, hydrochloride acid 32%, trichloroacetic acid, sodium metaperiodate, tert-butyl carbazate (tBC), ethylene glycol, hydroxylamine hydrochloride, trinitrobenzenosulfonic acid, methyl orange, McCoy’s 5A (Modified) Medium, l-glutamine, potassium bromide (KBr), inulinase and sodium bicarbonate were all acquired from Sigma–Aldrich. Sodium hydroxide was obtained from Ajax FineChem (Taren Point, New South Wale, Australia). CelluSep T4 25-mm flat width 12,000 MWCO Dialysis membranes were purchased from Fisher Biotec Australia (Wembley, Western Australia, Australia). Acetonitrile from Merck Pty Ltd. (Baywaters, Victoria, Australia). Deuterated water (D_2_O) and DMSO (DMSO-*d*_6_) for 1H NMR were obtained from Cambridge Isotope Laboratories (Tewksbury, MA, USA). For the MTT assay, 3-(4,5-dimethylthiazol-2-yl)-5-(3-carboxymethoxyphenyl)-2-(4-sulfophenyl)-2H-tetrazolium; 96-well plates; media ingredients including penicillin, streptomycin, and trypsin; and fetal bovine serum (FBS) were purchased from Thermo Fisher Scientific (Thebarton, Adelaide, Australia). All chemicals were of analytic grade and used as received without any further modification or purification.

### 2.2. Oxidation of Inulin to Inulin Aldehyde Derivative

#### Preparation and Characterization of Oxidized Inulin

Inulin was modified to obtain an aldehyde derivative by using the method previously reported [51]. Briefly, 1.2 g of inulin (12.3 mmol fructose units) was dissolved in 25 mL of distilled water, and a half equimolar amount of NaIO_4_ sodium periodate (0.6 g, 6 mmol) to fructose-repeating unit of inulin was added to the inulin suspension and stirred at 25 °C. The molar ratio of fructose-repeating units of raw inulin to NaIO_4_ was 2:1, which was expected to give a theoretical degree of oxidation (DO) of 50%. The periodate oxidation reaction was protected from light by carrying out the reaction in the dark and at room temperature. The reaction was allowed to proceed for different times (2 h, 3 h, 4 h, 15 h, and 20 h) in order to investigate the effect of reaction time on the oxidation process. The reaction was terminated by adding an excess quantity of ethylene glycol (molar ratio of 3:1 to NaIO_4_). Then, the reaction mixture was purified using a simple dialysis membrane bag in milliQ water for 3 days [52]. During the dialysis process, the water was changed daily, and the final oxidized inulin product (a white fluffy product) was obtained after freeze-drying the resulting solution.

### 2.3. Determination of the Degree of Oxidation (Aldehyde Content Analysis)

#### 2.3.1. Determination of Degree of Oxidation

The number of aldehyde functional groups obtained from the periodate oxidation reaction, referred to as the degree of oxidation, was quantified using hydroxylamine titration [53] and ^1^H NMR spectroscopy after reaction with tBC [41,54].

##### Hydroxylamine Titration

The reaction of hydroxylamine with oxidized inulin results in the formation of oximes and hydrochloric acid [53]. The released HCl was then quantified by titration with sodium hydroxide. For the hydroxylamine hydrochloride titration method, an accurately weighed amount of about 50.0 mg of each freeze-dried oxidized inulin sample was dissolved in 20 mL of 0.25 N hydroxylamine hydrochloride solution–methyl orange solution, and the pH was adjusted to 4.5 before use. The resulting mixture was allowed to react for 24 h with continuous stirring, resulting in the formation of oximes and hydrochloric acid. The released hydrochloric acid was then titrated with a standardized 0.5 mol/L NaOH solution, while the progress of the titration was monitored with a pH meter and visual observation of the color change until a red-to-yellow endpoint was achieved. The change in pH and the volume of sodium hydroxide consumed was recorded. Inulin oxidation using periodate produces two equivalent aldehyde groups for each mole of periodate consumed [51]. The related reactions and calculation formulas are as follows:

INU-(CHO)n + NH_2_OH·HCL → INU-(CH=N-OH)n + nH_2_O + nHCL

HCL + NaOH → NaCL + H_2_O.

The degree of oxidation can be calculated from the formula below:

(1) $$\;\frac{\mathrm{The}\;\mathrm{volume}\;\mathrm{of}\;\mathrm{NaOH}\;\mathrm{consumed}\;\times \mathrm{Concentration}\;\left(\mathrm{NaOH}\right)\;\times 10^{-3}}{\;\mathrm{Weight}\;\mathrm{of}\;\mathrm{the}\;\mathrm{sample}\;\left(g\right)\;\times \;\mathrm{Molecular}\;\mathrm{Weight}\;\mathrm{of}\;\mathrm{inulin}\;}=\frac{\;\mathrm{Mol}\;\mathrm{of}\;\mathrm{aldehyde}\;}{\mathrm{Mol}\;\mathrm{of}\;\mathrm{inulin}\;}$$

##### ^1^H NMR Spectroscopy after Titration with tBC

The degree of oxidation was also characterized using the ^1^H NMR spectroscopy method previously reported by Maia et al. [41]. In this method, a mixture of about 20 mg of oxidized inulin samples dissolved in about 3 mL of sodium acetate buffer with pH 5.0 (acidic condition) was reacted with excess tBC (81.278 mg) at room temperature for 24 h. Thereafter, the unreacted tBC was removed using dialysis. The resulting solution was then freeze-dried, and the ^1^H NMR spectroscopy spectrum was obtained using DMSO-*d*_6_ solvent. The ^1^H NMR spectroscopy method used in this experiment enables the quantification of the degree of oxidation (aldehyde content of the oxidized inulin) by comparing the ratio of the integral peak (δ) at 7.2 ppm belonging to the proton of the carbazone group to the glucose anomeric proton from inulin at δ 5.2 ppm. This method provides a similar result to that of colorimetric titration in the case of oxidized dextran. However, the formation of precipitates with highly oxidized sugar may limit the application of this method [41]. Using ^1^H NMR, the degree of oxidation of inulin can be determined using the equation below:

(2) $$\mathrm{The}\;\mathrm{degree}\;\mathrm{of}\;\mathrm{oxidation}\;=\frac{A\times 100}{B}$$

where A represents the integral peak (δ) at 7.2 ppm from the carbazone proton and B is the integral of the glucose anomeric proton at 5.2 ppm.

### 2.4. Preparation and Characterization of Hydrogels (Wissembourg, France)

The oxidized inulin (Oxi-4h) was dissolved in PBS (pH 7.4) to obtain a 6% (*w*/*v*) polysaccharide concentration [47]. AAD was also dissolved in PBS solution (pH 7.4) in order to obtain 2.5%, 5%, and 10% concentration. Finally, 800 µL of oxidized inulin solution was mixed with 200 µL of each different concentration of the AAD solution to form hydrogels [47]. The crosslinking of the hydrogels was allowed to proceed at 37 °C. Three hydrogels were formed using a different amount of crosslinker, namely INUAAD2.5, INUAAD5, and INUAAD10, corresponding to the gel obtained with 2.5%, 5% and 10% of crosslinker, respectively. The synthesized hydrogels were soaked in de-ionized water for 1 day in order to remove any unbound crosslinker and other unreacted reagents before freeze-drying.

### 2.5. Characterization of Oxidized Polymer and Hydrogels: FTIR and ^1^H NMR

The modification of the vicinal hydroxyl groups in inulin into aldehyde functional groups was confirmed by ^1^H NMR (Bruker Avance III 500 NMR, Wissembourg, France) and FTIR spectrophotometer (Shimadzu IRPrestige-21 FTIR 8400 spectrophotometer, Kyoto, Japan). The FTIR spectra of all the oxidized samples and hydrogels were acquired using a Schimadzu FTIR spectrophotometer in the region of 4000−400 cm^−1^ by recording 128 scans with a resolution of 4 cm^−1^. Briefly, about 2 mg of the freeze-dried samples and hydrogels were ground into powder before being mixed with about 100 mg of KBr and pressed into pellets using a hydraulic press. The ^1^H NMR spectra were acquired with a Brucker NMR spectrometer using a 5-mm broadband NMR probe, and DMSO was used as the solvent for all the samples except where stated otherwise.

### 2.6. Characterization of Hydrogels: TGA

Based on preliminary characterization, oxidized inulin obtained after 4 h of oxidation time was selected for further characterization and for hydrogel fabrication. About 6 ± 0.1 mg of each freeze-dried sample of oxidized inulin, crosslinker (AAD), and the hydrogels (INUAAD2.5, INUAAD5, and INUAAD10) were accurately weighed, and thermal gravimetric analysis was carried out in a TA Instrument (Thermogravimetric Analyzer Discovery TGA 550, New Castle, DE, USA) by heating the sample from 25 to 500 °C at a heating rate of 10 °C/min under a nitrogen atmosphere (10 mL min^−1^).

### 2.7. Characterization of Hydrogels: DSC

Differential scanning (DSC) of the synthesized inulin hydrogels (INUAAD2.5, INUAAD5, and INUAAD10), AAD, and the modified inulin was investigated using a Discovery DSC TA Instrument (model Discovery DSC 2920, New Castle, DE, USA). Briefly, ~2 ± 0.1 mg of each freeze-dried sample was weighed accurately in a platinum cup before being heated from 25 to 250 °C in a nitrogen atmosphere at a rate of 10 °C/min.

### 2.8. Rheological Evaluation

The rheological measurement of the synthesized inulin hydrogel was carried out using a rheometer (Malvern Kinexus pro+ Rotational, Worcestershire, UK) with parallel plates (diameter, 25-mm steel). In order to investigate the viscoelastic behavior of the hydrogels, the procured hydrogel was placed between the parallel plates with a gap of 1 mm in oscillatory mode while maintaining the temperature at 37 °C. Prior to rheological analysis, the inulin hydrogel was procured at 37 °C and allowed to stand for 30 min before testing. In order to ensure that all measurement was within the viscoelastic region, prior to the start of the frequency sweep experiments, a strain sweep was conducted using 1.0-Hz frequency and 0.1% strain. Thereafter, storage modulus (G′) and loss modulus (G′′) of the inulin hydrogels were determined using a frequency sweep test from 0.1 to 10 Hz and controlled strain (γ = 0.01).

### 2.9. Scanning Electron Microscope (SEM) Analysis

SEM imaging was conducted using FEI Quanta 450 FEG Environmental SEM (FEI, Hillsboro, OR, USA). Before the imaging process, the fully swollen hydrogels in sodium acetate buffer (pH 5.0) and PBS buffer (pH 7.4) were frozen using liquid nitrogen before freeze-drying to ensure that the morphology of the hydrogels was preserved. The freeze-dried hydrogels (INUAAD2.5, INUAAD5, and INUAAD10) were then transferred to double-sided tape before being sputter-coated with 10 mm of palladium, and the morphology was viewed using FEI Quanta 450 FEG Environmental SEM at an accelerated voltage of 10 KV. Furthermore, the pore size was determined from the SEM micrographs using image J software 1.49 v from the National Institutes of Health, Bethesda, MD, USA.

### 2.10. Dynamic Swelling Experiments: Swelling Analysis

The swelling properties of the hydrogels (INUAAD2.5, INUAAD5, and INUAAD10) were determined by the gravimetry method. Briefly, dry inulin hydrogel was weighed (Wd) before being immersed in deionized water and PBS buffer solution (pH 7.4) until the equilibrium swelling was reached, after which the hydrogels were removed. Excess surface water on the hydrogels was then blotted with the use of tissue paper before the swollen hydrogel was finally weighed. All the swelling experiments were performed in triplicate and at 37 °C. Finally, the equilibrium swelling ratio and percentage of swelling were calculated from Equations (3) and (4), respectively:

(3) $$\mathrm{Equilibrium}\;\mathrm{swelling}\;\mathrm{ratio}\;\mathrm{of}\;\mathrm{the}\;\mathrm{hydrogel}\;=\frac{\left(\mathrm{Ws}-\;\mathrm{Wd}\right)}{\mathrm{Wd}}$$

(4) $$\%S\;=\frac{\left(\mathrm{Ws}\;-\;\mathrm{Wd}\right)\times \;100}{\mathrm{Wd}}$$

where Ws represents the final weight of the fully swollen hydrogel and Wd is the dry weight of the hydrogel before swelling.

### 2.11. Release Kinetics of 5FU from Crosslinked Hydrogels

The encapsulation of 5FU to the hydrogels was accomplished during the crosslinking of the hydrogels. Briefly, 0.8 mL of oxidized inulin solution was mixed with 1 mg of 5FU before crosslinking this mixture with 0.2 mL of AAD, and subsequently, the mixture was incubated at 37 °C. The 5FU in the hydrogels was fixed at 1 mg/mL, and subsequently, the hydrogels were freeze-dried to obtain dried powder. Furthermore, the in vitro release experiment was carried out using the obtained freeze-dried powder. The release experiment was performed at physiological body temperature 37 °C in a 50-mL falcon tube. Then, 10 mL of release medium (phosphate buffer at pH 7.4 or sodium acetate buffer at pH 5.0) was added with constant stirring at 50 RPM. At scheduled time intervals, about 500 μL of the release aliquot was collected from the release medium, and the 5FU concentration in this aliquot was assayed using the HPLC method previously reported [55]. The HPLC system (Shimadzu Corporation, Kyoto, Japan) consisted of a pump (LC-20ADXR), an autosampler, a photodiode array (PDA) detector set to 268 nm, and a column for separation (Agilent Zorbax 300–SCX C18, 250 mm × 4.6 mm, 5 μm, Agilent Technologies Australia, Mulgrave, Victoria, Australia). The mobile phase for the HPLC assay was 10% acetonitrile and 90% water, and the flow rate was maintained at 0.9 mL/min in isocratic mode [55]. The injection volume of 5FU into the HPLC was 20 μL, the run time was 6 min, and the retention time for the 5FU peak was 3.061 min. The concentration of analytes was diluted to ensure that they fall within the standard calibration curve concentration range. A linear calibration curve of 5FU was plotted in the concentration range of 0.1–10 μg/mL (R^2^ > 0.999). All measurements were conducted in triplicate.

### 2.12. Degradation Studies

The degradation studies of the fabricated inulin hydrogels (INUAAD2.5, INUAAD5, and INUAAD10) were conducted in PBS (pH 7.4) without inulinase enzyme [49]. In addition, the in vitro degradation of the inulin hydrogels in the presence of inulinase was also investigated by immersing the three different hydrogels (INUAAD2.5, INUAAD5, and INUAAD10) into sodium acetate buffer (pH 5.0) containing 10 units inulinase/mL [36]. Inulinase was chosen because this enzyme is specifically expressed by the colon and is capable of degrading inulin polysaccharide [56,57,58]. In brief, dry inulin hydrogels (0.050 g) were added into 5 mL of the medium (PBS or acetate), followed by incubation at 37 °C with and without inulinase enzyme. At a predetermined time interval, the hydrogels were removed from the falcon tube and washed with water to remove any remnant of the enzyme. Finally, the remaining hydrogels were dried and weighed, and the weight was recorded as Wdt. All the experiments were performed in triplicate.

The degradation of the hydrogels was determined using Equation (5) below:

(5) $$\%\;\mathrm{Degradation}\;=\frac{\left(\mathrm{Wd}\;–\;\mathrm{Wdt}\;\right)\times 100}{\mathrm{Wd}}$$

where Wd and Wdt are the initial starting weights before and after degradation, respectively.

### 2.13. MTT Assay

The cytotoxicity of the synthesized inulin hydrogels and drug-loaded hydrogels were investigated against HCT116 human colorectal carcinoma cell lines (a gift from Professor Shudong of Wang Laboratory) using 3-(4,5-dimethylthiazol-2-yl)-2,5-diphenyl-tetrazolium bromide blue-indicator dye (MTT assay). First, HCT116 cancer cell lines were seeded in 96-well plates at 3 × 10^3^ cells per well, maintained under the expected standard growth condition (37 °C, 5% CO_2_, 10% FBS containing McCoy’s 5A (Modified) Medium), and incubated for 24 h to allow for proper cell attachment. Subsequently, the drug-loaded hydrogels and blank inulin hydrogels were sterilized for 15 min using an ultraviolet ray. This was followed by rinsing with sterile PBS. Then, 5 mL of McCoy’s 5A (Modified) Medium was added into the sterile hydrogel, and it was shaken for 24 h. Thereafter, the hydrogel was removed, and the resulting extracts were filtered using a 0.22-mm syringe. For the MTT assay, cells were incubated for 24 h with a free 5FU solution and the sterilized extracts from the hydrogels after diluting with McCoy’s 5A (Modified) Medium to obtain concentrations of 10, 50, and 100 µg/mL [59,60,61]. This incubation process was followed by washing with PBS, and then, the cells were further treated with 5 mg/mL of MTT solution at 37 °C for 4 h. The culture medium containing MTT reagent was removed, and 0.1 mL of DMSO was added. Finally, the absorbance of the resulting solution was measured at 570 nm using a microplate reader.

## 3. Results and Discussion

The vicinal hydroxyl groups on inulin were oxidized using sodium periodate, resulting in a highly reactive hemiacetal aldehyde derivative that subsequently reacted with adipic acid dihydrazide to form an inulin hydrogel via a hydrazone bond. Scheme 1 shows the oxidation of inulin by NaIO_4_ to create two aldehyde functional groups. Note that there is a chance of double oxidation from the glucose end group with the subsequent release of formic acid. However, this would likely represent a small portion of the polyaldehyde formed. This periodate oxidation occurred in an aqueous medium without utilizing any catalyst or any chemical initiator. The oxidation involved breaking the C3–C4 bond, resulting in two aldehyde groups [51], which then crosslinked with AAD to form a hydrogel. Prior studies suggest that one of the reactive aldehyde functional groups at C_3_ can undergo a reaction with the neighboring C_6_ hydroxyl group, which ultimately leads to the formation of stable intra-residue and inter-residue hemiacetals [62,63]. The stable hemiacetal could restrict the aldehyde functional groups available for nucleophilic reaction to one per fructose residue [51].

**Figure 1:**
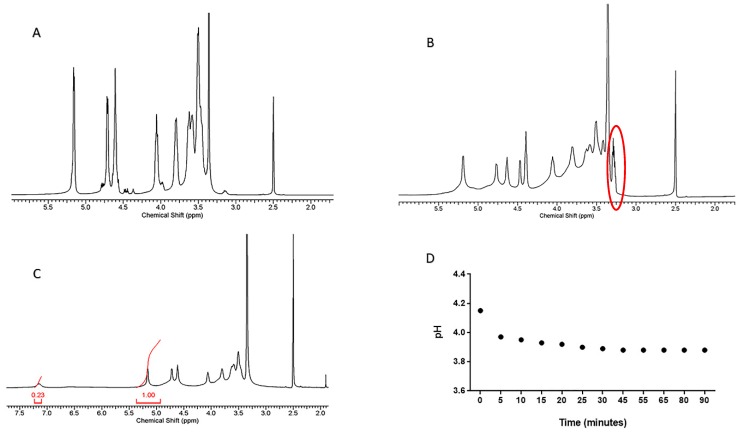
The ^1^H NMR spectra of (**A**) raw inulin, (**B**) oxidized inulin 2 h (Oxi-2h), and (**C**) oxidized inulin after titration with tert-butyl carbazate (tBC) using DMSO solvent; (**D**) the variation of pH during the oxidation of inulin with periodate. (Red circle show appearance of new peaks after oxidation).

During the early stages of the reaction (the first 10 min), an aldehyde functional group peak with very low intensity was observed around 9.5 ppm (Supplementary Materials Figure S1). This peak was not observed at the end of the reaction, possibly suggesting that a masked aldehyde group was involved in the formation of hemiacetals as the reaction progressed. The formation of hemiacetals was further supported by the appearance of a new peak in the ^1^H NMR spectra of the oxidized samples, with several distinctive peaks in the region of 3.5–5.0 ppm that were not present in the original raw inulin sample (Figure 1A,B). This result helps confirm that raw inulin was modified. (Supplementary Materials Figures S2 and S3A–E). The ^1^H NMR spectra of the oxidized inulin samples show visible change with the increase in time of the reaction from 2 to 20 h. (Supplementary Materials Figure S3A–E). The formation of hemiacetals, as shown in Figure 1B, is consistent with reports in the literature regarding some oxidized polysaccharides, such as dextran [41,64], cellulose [65], hyaluronic acid [48], alginate [66], starch, pectin, and chitosan [67]. There was rapid release of formic acid, possibly from the oxidation of the reducing end glucose as the reaction proceeded, resulting in a pH drop from 4.15 to 3.88; however, as the reaction proceeded the pH of the reaction medium remained stable at 3.88 for the remaining part of the reaction process (Figure 1D and Supplementary Materials Figure S1). The hemiacetals are behind the disappearance of the aldehyde functional group formed during oxidation.

### 3.1. Oxidized Inulin Characterization

The oxidized inulin derivative was also characterized using ^1^H NMR spectroscopy. From Figure 1C showing the ^1^H NMR spectrum of raw inulin, peaks between δ 3.0–4.80 ppm were assigned to fructose and glucose carbon-attached protons except for the anomeric proton from glucose that occurs at 5.2 ppm. The quantification of aldehyde groups, i.e., degree of oxidation (DO), was evaluated using tBC. The aldehyde content of the modified inulin was also investigated using ^1^H NMR spectroscopy by taking advantage of the reaction of the aldehydes with carbazate to form stable carbazones in same way as in hydrazone formation [41].

As shown in Figure 1C, the formation of carbazone shows that the reactive aldehyde portion of the oxidized inulin reacted with the amine portion of the tBC with evidence of a peak around 7.2 ppm. It is suggested that this peak resulted from the proton attached to the carbon atom modified by reacting the oxidized sample with tBC. Furthermore, there was evidence of the disappearance of peaks attributed to some of the hemiacetals after the formation of the final product. Comparing the carbazone integral’s peak δ at 7.2 ppm to that of the glucose anomeric proton at δ 5.2 ppm allowed DO quantification using ^1^H NMR spectroscopy (Figure 1A and Supplementary Materials Figure S5A–C). The DO of oxidized inulin (oxi-inu) 15 h and 20 h was not assayed by ^1^H NMR because of the slight oxidation of the anomeric glucose after the long oxidation period, making quantification difficult and inaccurate. Hydroxylamine titration converted the aldehydes into oximes with the release of acid quantified via a titrimetric method. The volume of sodium hydroxide at the endpoint was determined using the first derivative curve of the titration between sodium hydroxide and hydrochloric acid. The result of the ^1^H NMR spectroscopy for the oxidized inulin samples was lower than the result obtained from the hydroxylamine titration. The slight difference in the DO for the oxidized inulin samples (Table 1) can possibly be attributed to precipitation of the samples by the bulky tBC, as well as the pH of the reaction medium [51,68].

### 3.2. FTIR

**Figure 2:**
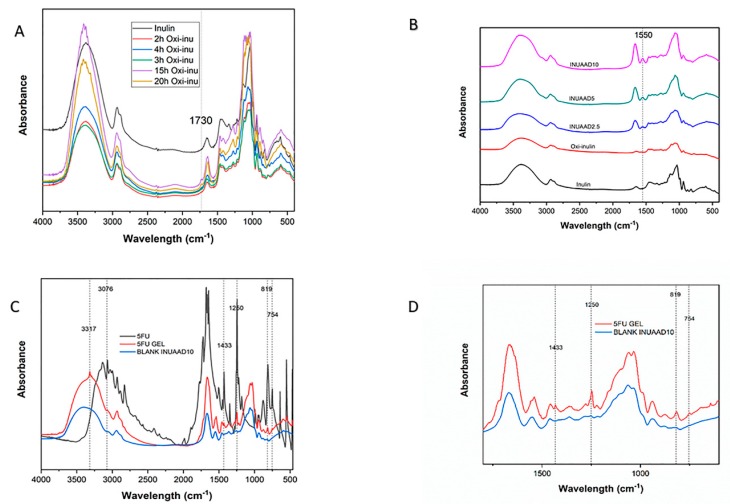
(**A**) The spectra of all the oxi-inulin and raw inulin samples. (**B**) The spectra of inulin, oxidized inulin (oxi-inulin), and the hydrogels. (**C**) Spectra of pure 5-fluorouracil (5FU), blank INUAAD10 gel, and 5FU-loaded hydrogel. (**D**) spectra of blank INUAAD10 and 5FU-loaded hydrogel.

In order to investigate the changes and alterations to the inulin structure as a result of the periodate oxidation, both the raw inulin and modified samples were analyzed using FTIR. Despite the oxidation reaction, the inulin structure was partly preserved. Figure 2A shows the FTIR spectra of the raw inulin and modified inulin samples with different degrees of oxidation based on the reaction time. In Figure 2A, it is difficult to see any difference between the oxidized and raw inulin. However, for the highly oxidized inulin samples (oxi-15h and oxi-20h) there was a low-intensity signal at 1730 cm^−1^ ascribed to the stretching of the carbonyl group from an aldehyde [69]. In addition, there were several changes in the position of the bands, especially in the region of 1300–800 cm^−1^ for the oxidized inulin compared with the raw inulin (Supplementary Materials Figure S6A). The formation of hemiacetals made it difficult to notice any aldehyde groups in the oxidized sample [41], and this is consistent with the reported FTIR of oxidized polysaccharides, such as dextran [41] and alginates [52,70].

The formation of the hydrogels by crosslinking oxidized inulin with AAD was confirmed by FTIR spectroscopy (Figure 2B). The spectra of the hydrogels show the appearance of the new band at 1550 cm^−1^, which can be attributed to the NH bending from the crosslinker (AAD). The 5FU-loaded inulin hydrogels were also characterized using FTIR (Figure 2C,D). The FTIR spectra of the 5FU-loaded inulin hydrogels reveal small changes compared with the blank inulin hydrogels, including new bands at 3076, 1433, 1246, 819, and 754 cm^−1^ [71], which were attributed to the incorporation of 5FU. Furthermore, the change in the broad peak of the blank gels compared with that of the 5FU-loaded gels was presumably due to the hydrogen bonding interaction between the OH of the hydrogel and the NH stretching of 5FU, resulting in the formation of a sharp peak around 3317 cm^−1^ [71,72] (Figure 2C). The disappearance of some typical 5FU peaks can also be attributed to the strong hydrogen bond interactions between the blank gels and 5FU (Figure 2C,D and Supplementary Materials Figure S6B,C).

### 3.3. SEM

**Figure 3:**
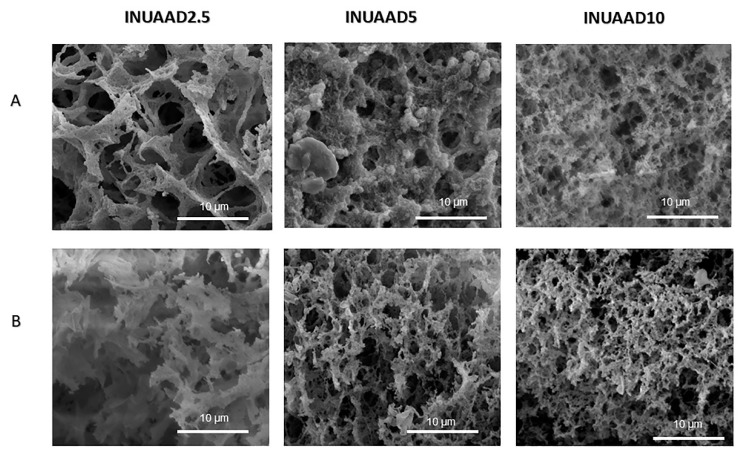
(**A**) SEM images of hydrogels (INUAAD2.5, INUAAD5, and INUAAD10) after immersion in acetate buffer for 2 days. (**B**) SEM images of hydrogels (INUAAD2.5, INUAAD5, and INUAAD10) after immersion in phosphate-buffered saline (PBS) for 2 days.

The interior morphologies of the fabricated inulin hydrogels, as well as their porous structures, were evaluated by SEM. Figure 3A,B clearly shows that the freeze-dried hydrogels (INUAAD2.5, INUAAD5, and INUAAD10) had irregular, interconnecting inner pores. It is evident that the pore size of the hydrogels decreased with the increase in AAD, as higher concentrations of AAD resulted in higher crosslink density, producing gels with smaller pore size. SEM was also able to show the pore size variation and hydrolysis of the hydrazone bond when the gels were immersed in acetate buffer (pH 5.0) as compared with PBS (pH 7.4) for 2 days. The SEM images confirm the slight degradation of the hydrogels in acetate buffer due to the hydrolysis of the bonds, as well as the increase in pore size (Figure 3A). The formation of porous materials has also been reported for other oxidized polysaccharides crosslinked with AAD in the literature [41,50], and pore formation with controllable pore size is important for drug encapsulation and delivery of drugs such as 5FU.

### 3.4. TGA/First derivative of the TGA Curve (DTG)

**Figure 4:**
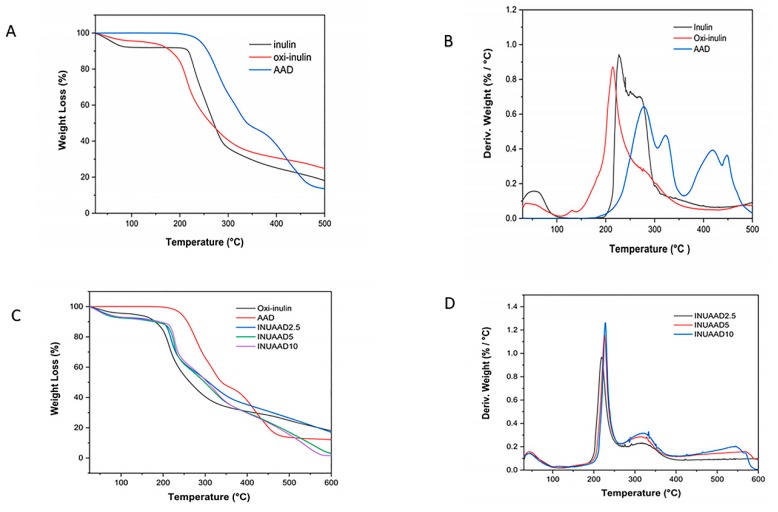
(**A**) Thermograms of the raw inulin, oxidized inulin, and AAD. (**B**) DTG thermograms of the raw inulin, oxidized inulin, and AAD. (**C**) Thermograms of the raw inulin, oxidized inulin, AAD, and hydrogels. (**D**) DTG thermograms of the hydrogels.

The thermal stability of the raw inulin, oxidized inulin, AAD, and hydrogels was assessed using TGA/DTG. As shown in Figure 4A,B, the crosslinker showed higher thermal stability compared with oxidized inulin. The TGA curve for AAD shows an onset of degradation at 282 °C, and raw inulin showed slight weight loss due to the elimination of water at a temperature below 100 °C and major weight loss occurring between 225 and 325 °C, which was attributed to inulin backbone degradation [73]. There was a clear shift in the onset temperature for degradation from 215 °C for raw inulin to 192 °C for oxidized inulin (reduced by 25 °C), and there was a different character of the thermogram that can be attributed to the damage to the raw inulin chain caused by its oxidation [74,75,76,77]. After crosslinking the oxidized inulin with AAD, the thermal stability of the synthesized hydrogels was enhanced and improved compared with the oxidized inulin, as seen in the TGA and DTG curve (Figure 4C). This was due to formation of a stable hydrazone bond and strong hydrogen bonding interactions introduced onto the molecular chain [78,79,80]. As expected, the hydrogels exhibited three stage mass loss. Below 100 °C, there was the removal of bound and free water in the hydrogels (about 7% weight loss). The second weight loss was due to the decomposition of the inulin backbone, and finally, the third stage was due to the degradation of the AAD. Increasing the AAD concentration during crosslinking resulted in hydrogels with higher decomposition temperature. The maximum thermal decomposition temperature for the hydrogels was found to be 218.4, 225.9, and 227.5 °C for the three hydrogel samples, INUAAD2.5, INUAAD5, and INUAAD10, respectively. The corresponding residual weight at 600 °C for the INUAAD2.5, INUAAD5, and INUAAD10 hydrogels was 17.2%, 3.02%, and 1.55% respectively. It is important to mention that all the oxi-inu hydrogels showed a similar degradation profile (Figure 4D). Proof of crosslinking between the modified oxidized inulin and AAD was confirmed by the change in the maximum temperature of degradation of the hydrogels when compared with that of the oxidized inulin.

### 3.5. DSC

**Figure 5:**
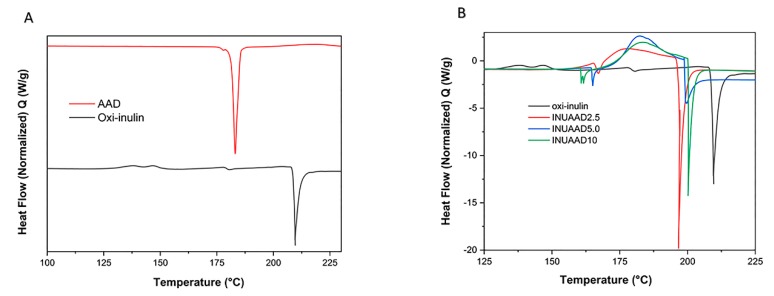
(**A**) Differential scanning calorimetry (DSC) thermograms of the oxidized inulin and AAD; (**B**) thermograms of the oxidized inulin and hydrogels.

The DSC of the oxidized inulin, AAD, and hydrogels was also investigated (Figure 5A,B). As shown in the DSC thermogram, the melting point peak of the oxidized inulin was 161.3 °C, and the melting point of AAD was 183.22 °C. However, these peaks were absent in the final hydrogels, indicating the formation of new crosslinked polymeric material. In Figure 5B, the hydrogels exhibited endothermic peaks at 166, 164, and 160 °C for INUAAD2.5, INUAAD5.0, and INUAAD10 respectively, due to degradation of the hydrazone bond [16,81]. Interestingly, as the amount of AAD increased, the temperature of the endothermic peak decreased. In addition, there was a broad exothermic transition for all the hydrogels after the degradation of the hydrazone bond. Another endothermic peak at 196.96, 199.42, and 200.26 °C for INUAAD2.5, INUAAD5.0, and INUAAD10, respectively, can be seen in Figure 5B, which was attributed to the start of the hydrogel degradation. It is very clear that the degradation temperature was dependent on the crosslinking density.

### 3.6. Rheological Properties

**Figure 6:**
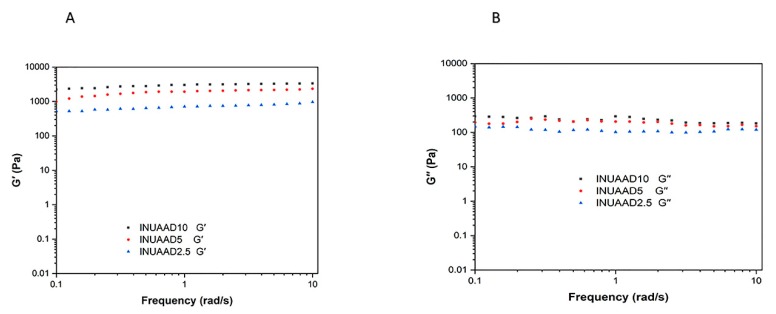
(**A**) Rheological properties of the INUAAD hydrogels showing the storage moduli (G′) and (**B**) the loss moduli (G”) of the different crosslinked inulin hydrogels.

As shown in Figure 6A,B, all three fabricated inulin hydrogels, INUAAD2.5, INUAAD5, and INUAAD10, showed higher G′ compared with the G′′ for all the frequencies tested using the frequency sweep mode. As shown in Figure 5A, it is obvious that the G′ increased with the increase in the concentration of the crosslinker from 2.5% to 10%, whereas there was little change with respect to G′′. By increasing the crosslinker from 2.5% to 10%, the G′ increased from ca. 711 to 3015 (Pa) at a frequency of 1 Hz (Supplementary Materials Table S1). This increase in G′ indicates that a stiff and highly crosslinked gel was formed at high AAD concentration [48]. These results indicate that the fabricated hydrogels possessed viscoelastic properties [52,82]. All the hydrogels exhibited high G’ value, which was independent of the frequency that is indicative of a gel with stable viscoelastic solid-like behavior [52]. In addition, all the synthesized inulin hydrogels had similar loss moduli. The difference in elastic moduli also shows that the crosslinking density for the gels differed. This result corroborates the finding that, by varying crosslinker concentration, we can synthesize hydrogels with different degrees of crosslinking. The rheology measurements, particularly the elastic moduli of all the hydrogels, as well as the crosslink density, had a direct correlation to the content of AAD used during crosslinking.

### 3.7. Gelation and Swelling Studies

The swelling ratio of the fabricated inulin hydrogels in de-ionized water and PBS (pH 7.4) (Supplementary Materials Figure S7) shows a higher swelling ratio (SR) in de-ionized water compared with PBS, as expected. The gels showed moderate swelling in water, with a swelling ratio of 6.95, 5.61, and 3.8 for INUAAD2.5, INUAAD5, and INUAAD10, respectively. The result demonstrates that the swelling ratio of the hydrogels was inversely related to the degree of crosslinking. Furthermore, all the hydrogels reached equilibrium before 24 h, which can help facilitate the diffusion and release of drug molecules from such hydrogels. The SR obtained was comparable to pH-sensitive inulin hydrogels with reported values from 3.5 to 6.5 [19,37] but lower than inulin hydrogels made from pyromellitic dianhydride crosslinker [16]. The SR was also lower than the values reported for oxidized dextran crosslinked with AAD (4.3–8.7) [41] and polyaldehyde guluronate (12.6–15.3) [69]. The gelation time for the fabricated hydrogels obtained by the tube inversion method was directly related to the concentration of AAD used during hydrogel synthesis. The gelation time was reduced from 178 to 128 s with an increase in AAD concentration, which was very close to those reported for oxidized hyaluronic acid/ adipic acid dihydrazide hydrogels (143–175 s) [48] and oxidized dextran/AAD gels (117–230 s) [41]. By controlling the amount of AAD used, we can easily tune the gelation time. As the concentration of AAD used during the hydrogel fabrication increases, the time required for the formation of the hydrogel decreases, which is attributed to the higher percentage of hydrazide available for effective crosslinking.

### 3.8. Release Kinetics of 5FU from Crosslinked Hydrogels

**Figure 7:**
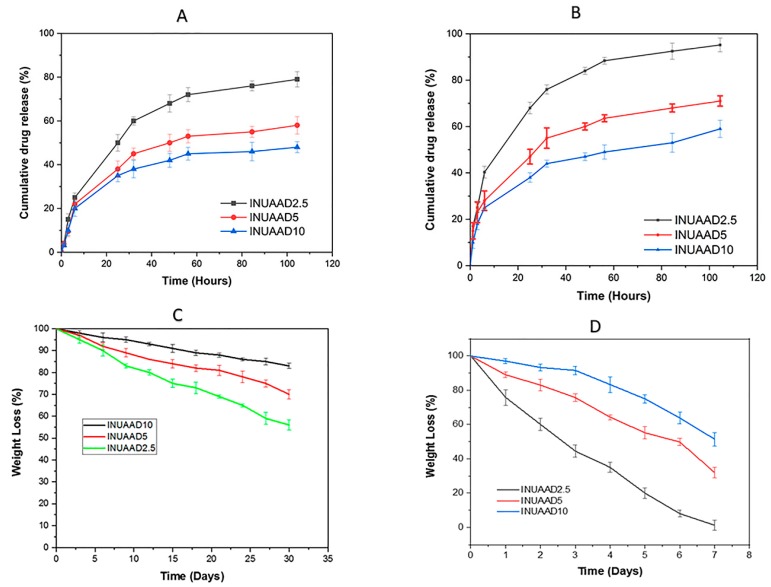
(**A**) The in vitro release rate of 5FU from the hydrogel batches in pH 7.4; (**B**) the in vitro release rate of 5FU from the hydrogel batches in pH 5.0; (**C**) the degradation of the hydrogels in pH 7.4; (**D**) the degradation of the hydrogels in pH 5.0 with the addition of inulinase.

The drug delivery capability of the fabricated inulin hydrogels was further evaluated using 5FU as the model drug for in vitro release. The in vitro release results show that the amount of 5FU released can be tuned by varying the crosslinker ratio. The release of 5FU was initially characterized by burst release, which was subsequently followed by sustained release, as expected. In this study, the hydrogels with hydrazone linkages allowed faster cleavage at low acidic pH. The inulin hydrogel system exploited acidic pH around colon cancer tissues to achieve better drug release properties. Overall the release profile of 5FU from the crosslinked hydrogels was governed by both the pH of the release medium and the crosslinking density. As shown in Figure 7A,B, as expected, all the hydrogels demonstrated faster drug release in pH 5.0 than in pH 7.4. The hydrazone bond of the inulin hydrogels was relatively stable in physiological pH but was cleaved in acidic pH. As shown in Figure 7A,B, there was a burst release in both pH 5 and 7.4 on the first day, which was followed by a very slow release of 5FU. In the release study, 50%, 38%, and 35% of 5FU was released from INUAAD2.5, INUAAD5.0, and INUAAD10, respectively, in pH 7.4 after 24 h. In contrast, in the acidic media with pH 5.0, after 24 h, 68%, 47%, and 38% of 5FU was released from INUAAD2.5, INUAAD5.0, and INUAAD10, respectively. Moreover, after the initial burst release in pH 5.0, there was a continuous release of 5FU, with about 85% of 5FU released within 2 days for INUAAD2.5. The gels showed higher release in pH 5.0 compared with physiological pH 7.4.

The release of 5FU from the hydrogels was dependent on the crosslinking density and pH of the release media. The FTIR result for the 5FU-loaded hydrogels show that the 5FU amine group and inulin polymer hydroxyl may have formed a hydrogen bond interaction [71,83,84]. This polymer–drug interaction may have resulted in slow drug release from the hydrogel due to entrapment of the remaining drug in the gel network (Figure 2C,D). The smaller pore size allowed the encapsulation of structurally smaller drug moieties, such as 5FU, into the hydrogels [71]. The difference in the stability of the hydrazone bond in pH 7.4 compared with pH 5.0 was exploited for selective and localized targeting of 5FU between colon normal and cancers cells. The difference performance with respect to pH enables the use of this hydrogel as a useful strategy for delivering 5FU to the colon. This result clearly suggests that the acidic environment within colon cancer cells would trigger the degradation of the crosslinked hydrogel, which would result in a higher drug release from the hydrogel. This hydrogel system can potentially maintain a higher concentration of drugs around cancer cells and can possibly reduce adverse side effects on healthy tissue. In order to achieve successful colon targeting, the inulin drug delivery system will require enteric coating to prevent gastric degradation and premature release of drugs as the hydrogel passes through the stomach and small intestine.

### 3.9. Degradation

The degradation rate of the hydrogels was investigated by examining the weight loss in both PBS and acetate buffer, as shown in Figure 7C,D. INUAAD10, INUAAD5.0, and INUAAD2.5 experienced weight loss of 17%, 30%, and 44%, respectively, in PBS after 30 days of incubating, implying that a higher concentration of the crosslinker results in higher resistance to biodegradability. In addition, the in vitro degradation of the inulin hydrogels in the presence of inulinase incubated in sodium acetate buffer (pH 5.0) after one week was 49%, 68%, and 95% degradation for INUAAD10, INUAAD5, and INUAAD2.5, respectively (Figure 7D). Inulinase is known to degrade inulin in the colon and function better around pH 4.7, which is close to pH 5.0 [56]. As shown in Figure 7C,D, the degradation rate decreased with the increase in AAD concentration. This finding could be attributed to the increase in degree of crosslinking, making it harder for the inulinase to attack the hydrogel, as well as the higher hydrazone bond, which tends to be resistant to hydrolytic cleavage. The degradation behavior in acetate buffer was related to the cleavage of the hydrazone bond due to the acidic pH of the medium, as well as partial activity from the enzyme. The hydrogels’ degradation was pronounced in acetate buffer with inulinase but slow in physiologic pH conditions. The in vitro degradation data clearly show that it is possible to tune the degradation rate by varying the ratio of the crosslinker. The prolonged degradation profile of these hydrogels make them useful for controlled delivery of drugs to the colon.

### 3.10. Cytotoxicity

**Figure 8:**
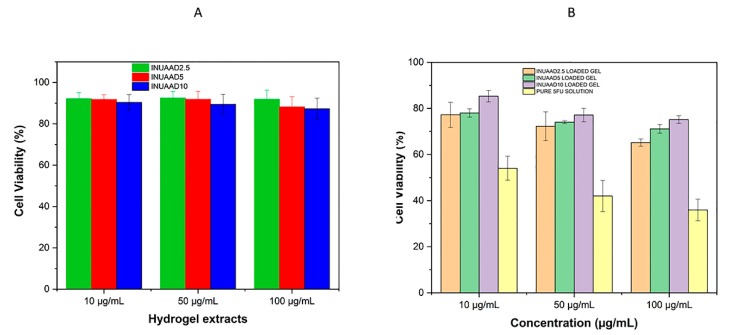
(**A**) Cell viability of the HCT116 cancer cells after treatment with the blank hydrogel extracts, and (**B**) cell viability of HCT116 cancer cells after treatment with the 5FU-loaded hydrogel extracts.

The blank crosslinked inulin hydrogels were assayed against the HCT116 cancer cell line using MTT assay, because the application of the synthesized inulin hydrogel as a drug delivery carrier requires the biomaterials to be non-toxic and the 5FU-loaded inulin hydrogels were expected to cause high cytotoxicity to tumor cells. The cell cytotoxicity of different concentrations of extracts from the crosslinked inulin hydrogels, oxidized inulin, and crosslinker was evaluated. As shown in Figure 8A, the viabilities of HCT116 with different extracts of empty inulin hydrogel samples were all above 85% after 24 h of incubation, which clearly demonstrates that these hydrogels were biocompatible with negligible cytotoxicity at concentrations between 10 and 100 μg/mL and that they can be utilized as a delivery platform to deliver drugs to the colon.

In Figure 8A, we can clearly observe, as expected, a dose-dependent decrease in the cell viability as the concentration of the sample increased from 10 to 100 μg/mL with respect to the hydrogel extracts. The blank crosslink hydrogels did not have any appreciable cytotoxicity towards the HCT116 cancer cells. As expected, after 24 h of incubation, both the modified inulin with the aldehyde functional group and AAD showed reduced cell viability with a viability of 77.1% and 88%, respectively (Supplementary Materials Figure S8). This can be attributed to the reactive aldehyde group in the oxidized inulin, which tends to react to the nucleophilic portion of the cells. In addition, the 5FU-loaded hydrogels were assayed for its antitumor activity against the HCT116 cancer cells via MTT assay, and the free 5FU solution was employed as a control. After 24 h of incubation, the 5FU-loaded hydrogels showed a dose-dependent antitumor effect. Furthermore, the gel with the lowest concentration of crosslinker swelled better in the medium, causing a greater release of 5FU and more activity. As shown in Figure 8B, the cell viability of the 5FU-loaded gels against the HCT116 cell line was 65.2%, 71.1%, and 75.2%, respectively, compared with free 5FU, which was 35.9% at 100 μg/mL concentration. The difference in cell viability between the pure and loaded hydrogels was due to the slow release of 5FU from the hydrogel structure. The free 5FU exhibited more antitumor activity compared with the 5FU-loaded hydrogels. This was expected, because the fabricated hydrogel releases 5FU slowly as and a pH of 7.0 does not break the hydrazone bond of the gel. These results support the finding that inulin hydrogel could be utilized as a delivery platform. Based on these results, we can conclude that extracts obtained from the blank inulin hydrogels did not show any significantly difference from the control in terms of cell viability.

## 4. Conclusions

New injectable inulin hydrogels based on hydrazone crosslinking for potential use as drug delivery vehicles were fabricated by reacting oxidized inulin with AAD without the use of a catalyst. The novel hydrogels were formed within 2–3 min at 37 °C. The initial degree of oxidation was determined by hydroxylamine titration and ^1^H NMR spectroscopy, whereas FTIR spectroscopy was used to confirm the covalent crosslinking by AAD with a new band at 1550 cm^−1^. Changes in thermal properties was detected using DSC and TGA. By varying, the amount of AAD crosslinker used during hydrogel synthesis, the swelling, rheological properties, pore size, degradation, and 5FU release of the hydrogels can be tuned. The in vitro release rate of 5FU from all the hydrogels tested exhibited initial burst release followed by a controlled release pattern. The hydrogels are degradable both hydrolytically in physiologic conditions and by inulinase. In addition, the MTT results confirm that, at a concentration of 10–100 µg/mL, the INUAAD hydrogels showed no appreciable cytotoxicity. These findings demonstrate that these novel hydrogels could serve as delivery systems for anticancer drugs in the treatment of colon cancer.

## Figures and Tables

**Table 1:** The aldehyde content determination using ^1^H NMR spectroscopy and hydroxylamine titration.

Oxidized Inulin	The Degree of Oxidation by Hydroxylamine (%) ^a^	The Degree of Oxidation by NMR (%) ^b^
**Oxi-2h**	25.6 ± 0.6	23 ± 0.9
**Oxi-3h**	31 ± 0.8	26 ± 0.7
**Oxi-4h**	34 ± 0.3	31 ± 1.2
**Oxi-15h**	38 ± 0.4	^n^
**Oxi-20h**	43.6 ± 0.5	^n^

^a^ The results of all titration experiments were the average of three titrations; ^b 1^H NMR results calculated from four independent integrations with Advanced Chemistry Development (ACD) software; ^n^ not estimated.

## Supplementary Materials

The Supplementary Materials are available online at https://www.mdpi.com/1999-4923/11/7/356/s1.
