# Supplementary material for: Preparation and Characterization of Oxidized Inulin Hydrogel for Controlled Drug Delivery
Source: Pharmaceutics. 2019 Jul 22;11(7):356. doi: 10.3390/pharmaceutics11070356 (PMC6680939; doi:10.3390/pharmaceutics11070356)
Supplement: Supplementary file 1 [file pharmaceutics-11-00356-s001.pdf]

pharmaceutics

MDPI

Supplementary Materials: Preparation and Characterization of Oxidized Inulin Hydrogel for Controlled Drug Delivery

Franklin Afinjuomo, Paris Fouladian, Ankit Parikh, Thomas G. Barclay, Yunmei Song and Sanjay Garg

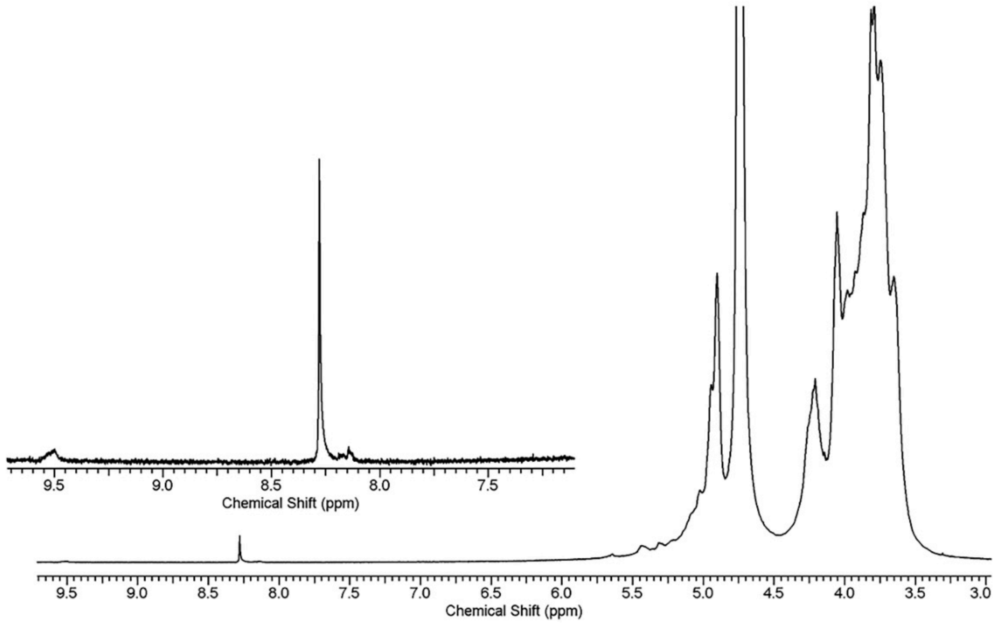

Figure S1. ^1H NMR spectra of inulin oxidation with low-intensity peak of aldehyde at 9.5 ppm as well as peak due to formic acid around 8.25 ppm as the reaction proceed in D_2O.

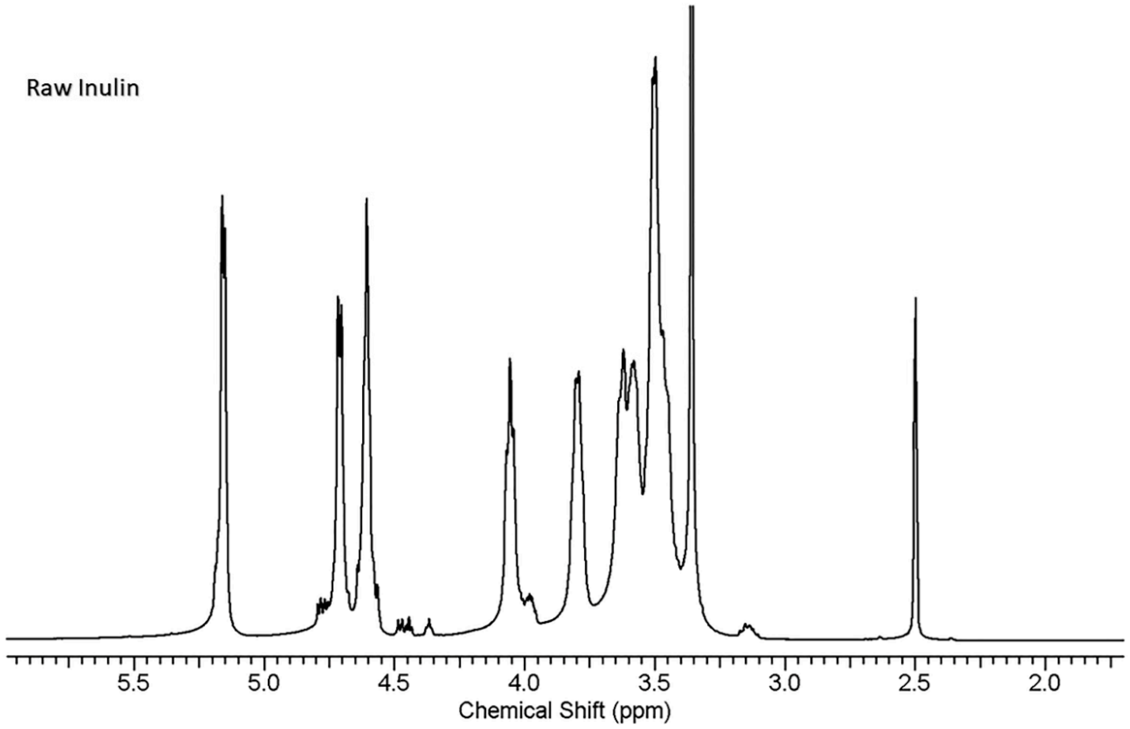

Figure S2. ^{1}H NMR spectra of raw unmodified inulin in deuterated DMSO solvent.

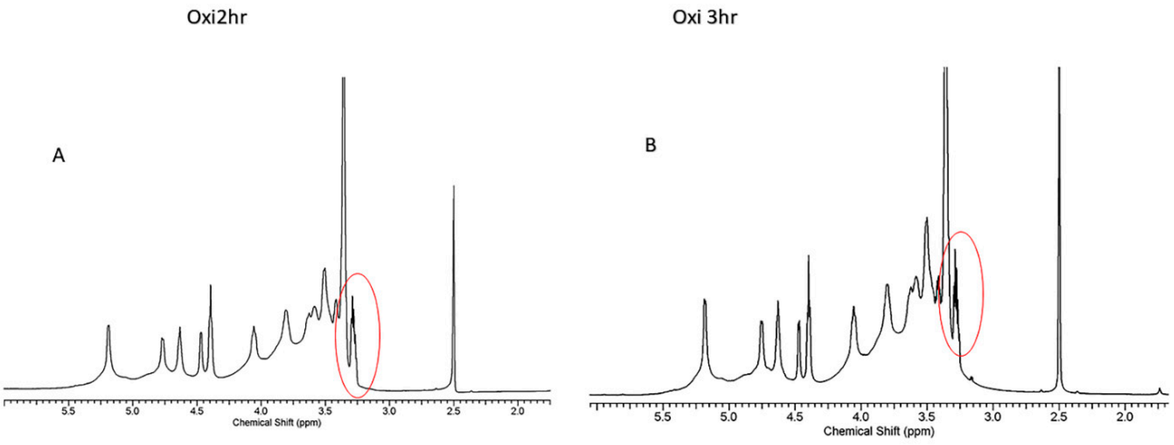

Figure S3A and B. ^{1}H NMR spectra of oxidized inulin (**A**) 2 h and (**B**) 3 h in deuterated DMSO Solvent.

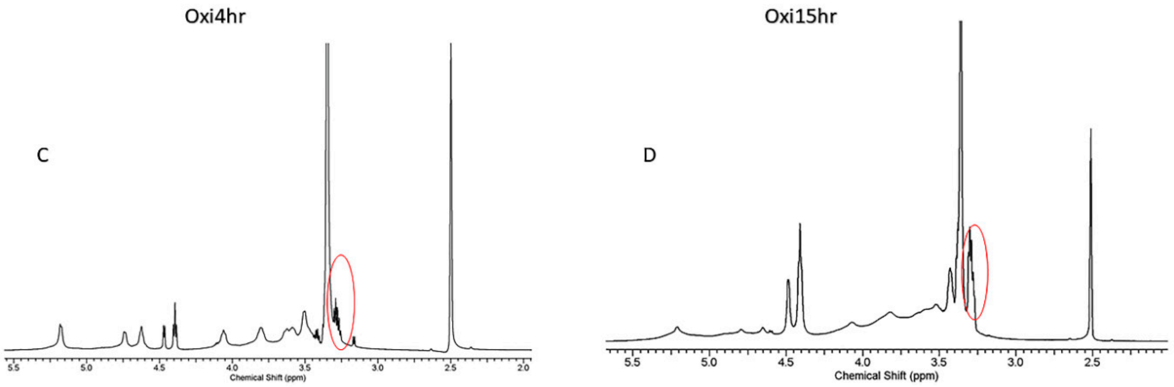

Figure 3C and D. ^{1}H NMR spectra of oxidized inulin (**C**) 4 h and (**D**) 15 h in deuterated DMSO solvent.

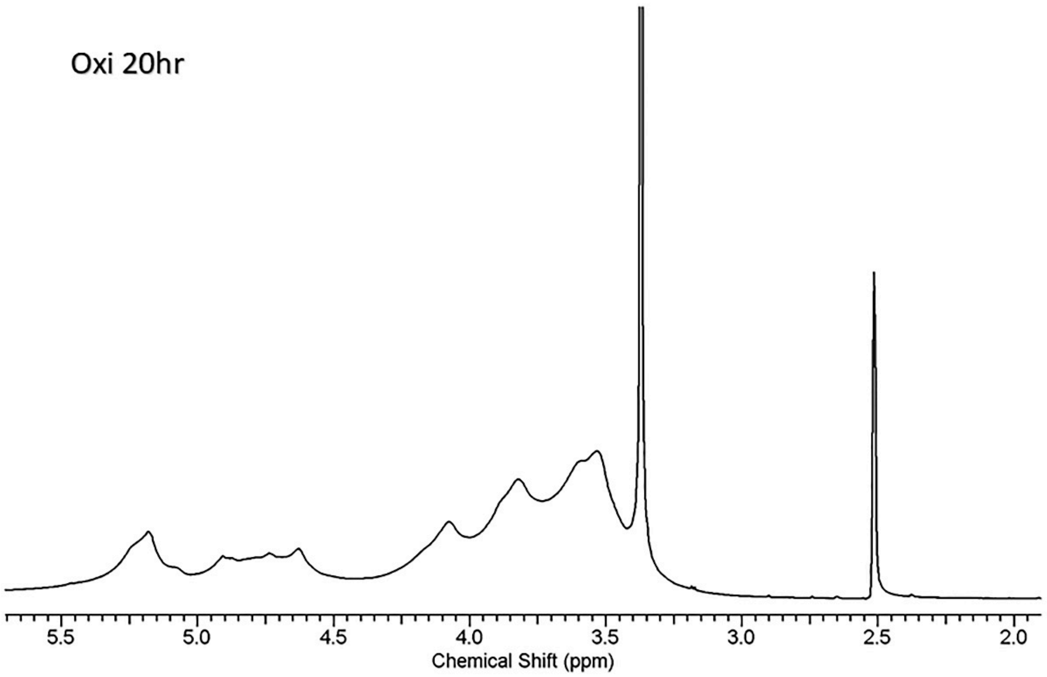

Figure S3E. ^{1}H NMR spectra of oxidized inulin 20 h in deuterated DMSO solvent.

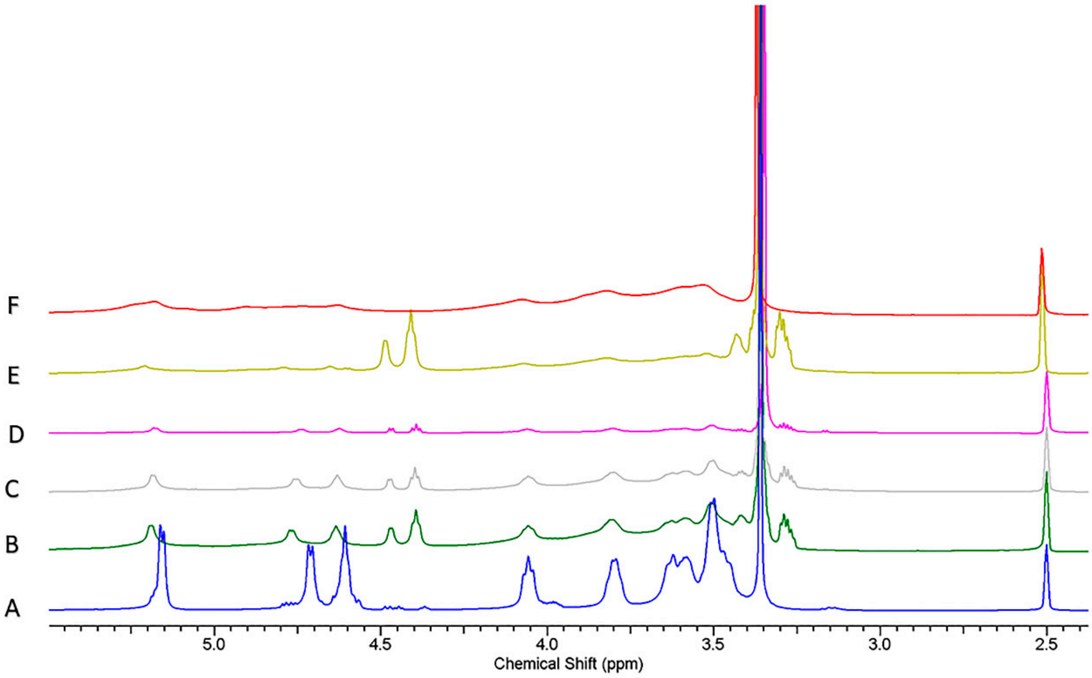

Figure S4. ^{1}H NMR spectra of both raw inulin and all oxidized samples in deuterated DMSO solvent: (**A**) raw inulin and oxidized inulin (**B**) 2 h, (**C**) 3 h, (**D**) 4 h, (**E**) 15 h, and (**F**) 20 h.

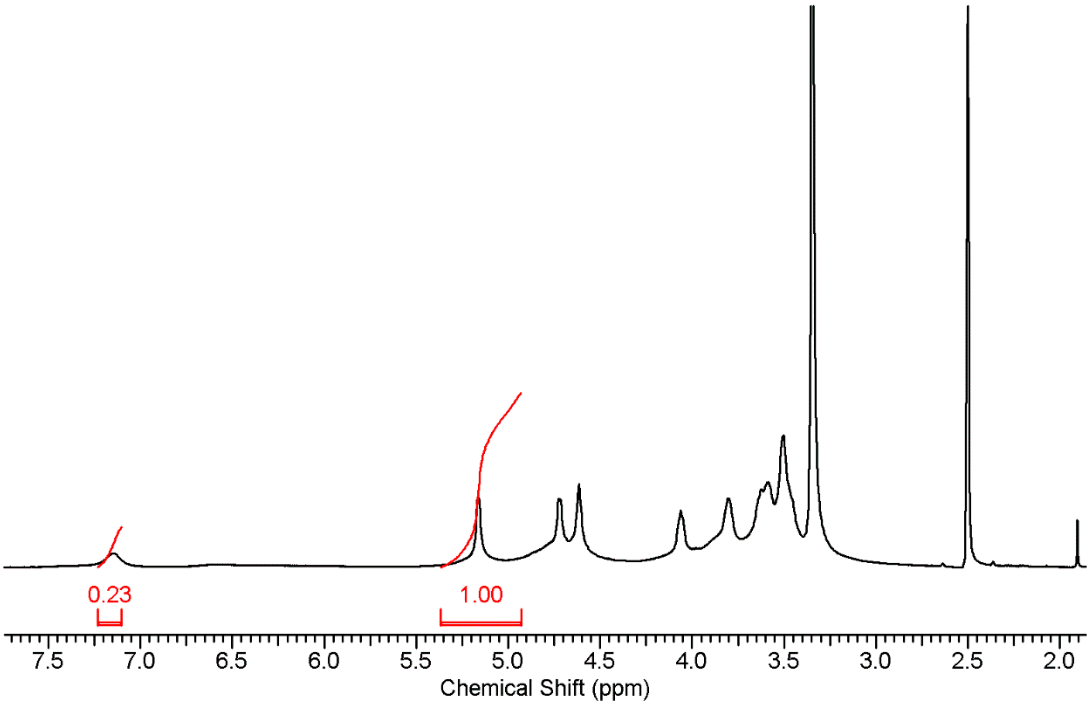

Figure S5A. ^{1}H NMR spectra of OXI2hr reacted with tBC for the determination of DO.

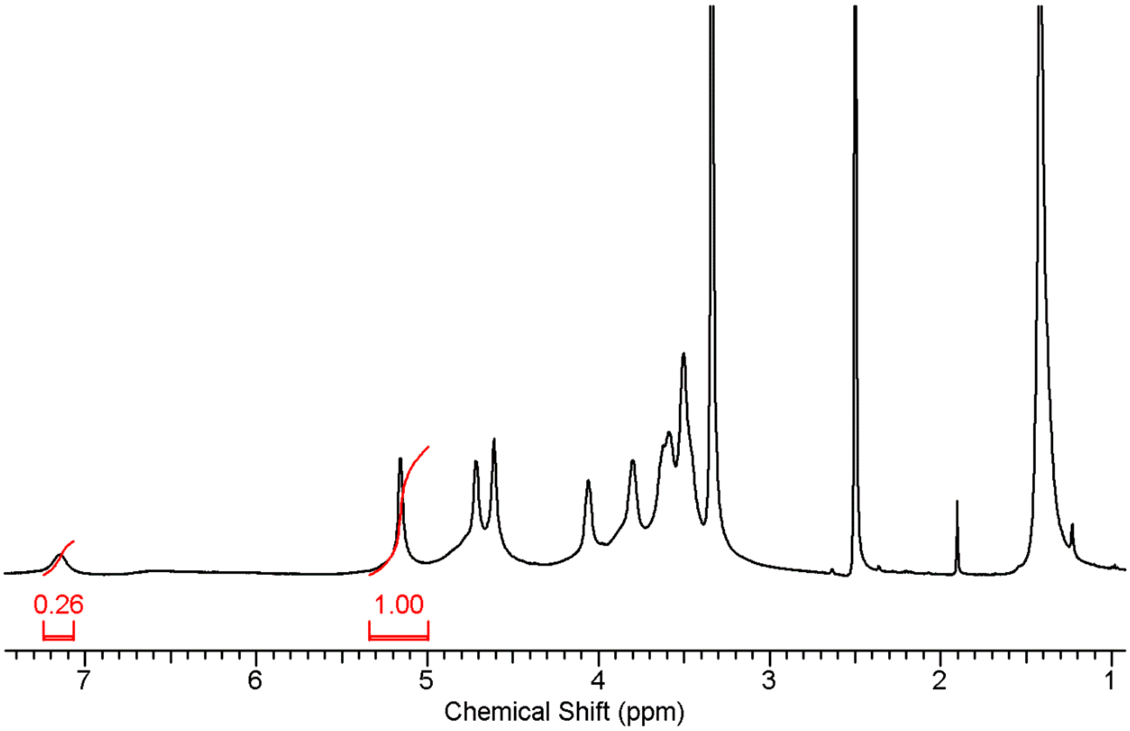

Figure S5B. ^{1}H NMR spectra of oxi-3h reacted with tBC for the determination of DO.

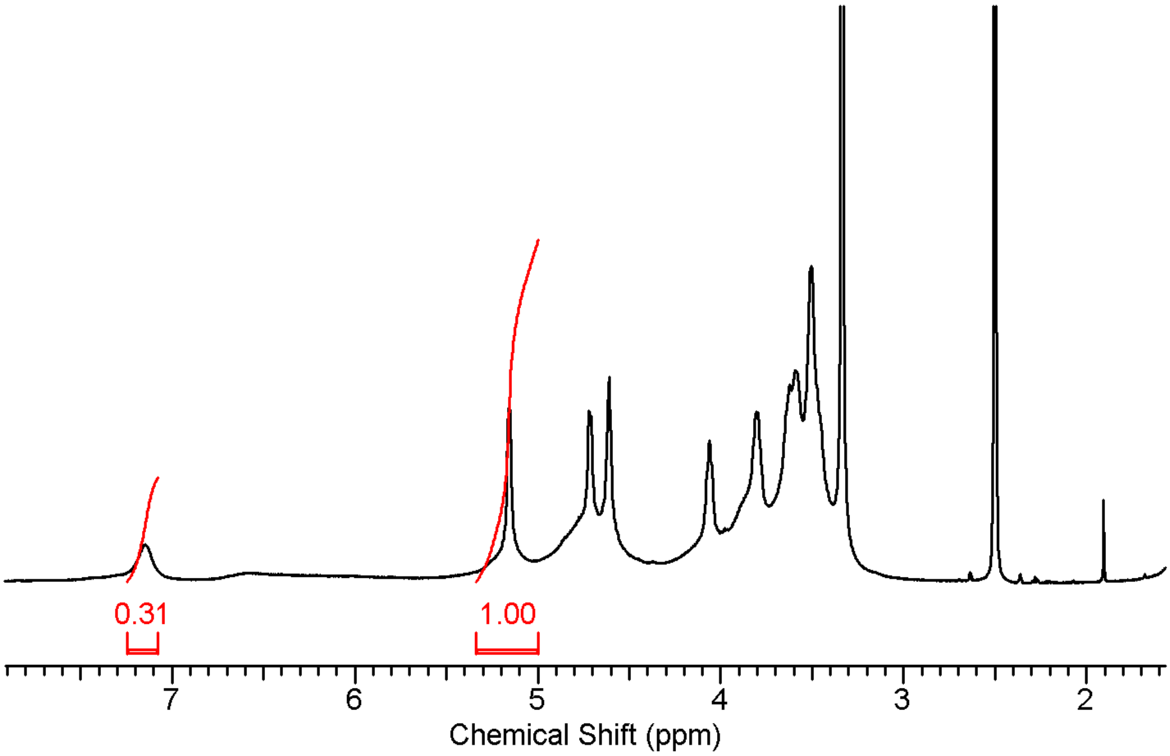

Figure S5C. ^{1}H NMR spectra of oxi-4h reacted with tBC for the determination of DO.

FTIR

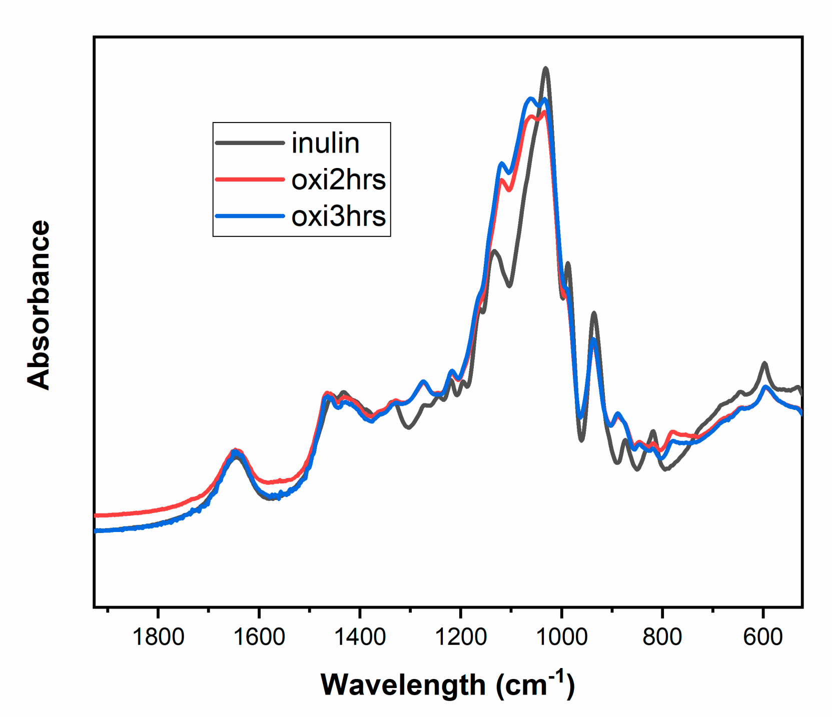

Figure S6A. Slight shift in the FTIR spectra peak for the modified inulin in comparison to raw inulin.

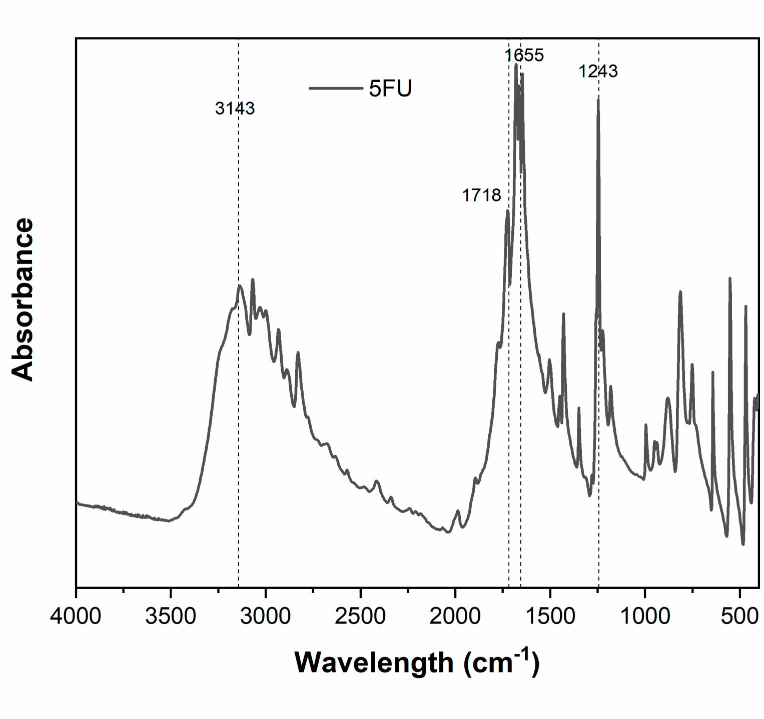

Figure S6B. FTIR spectra of pure 5FU.

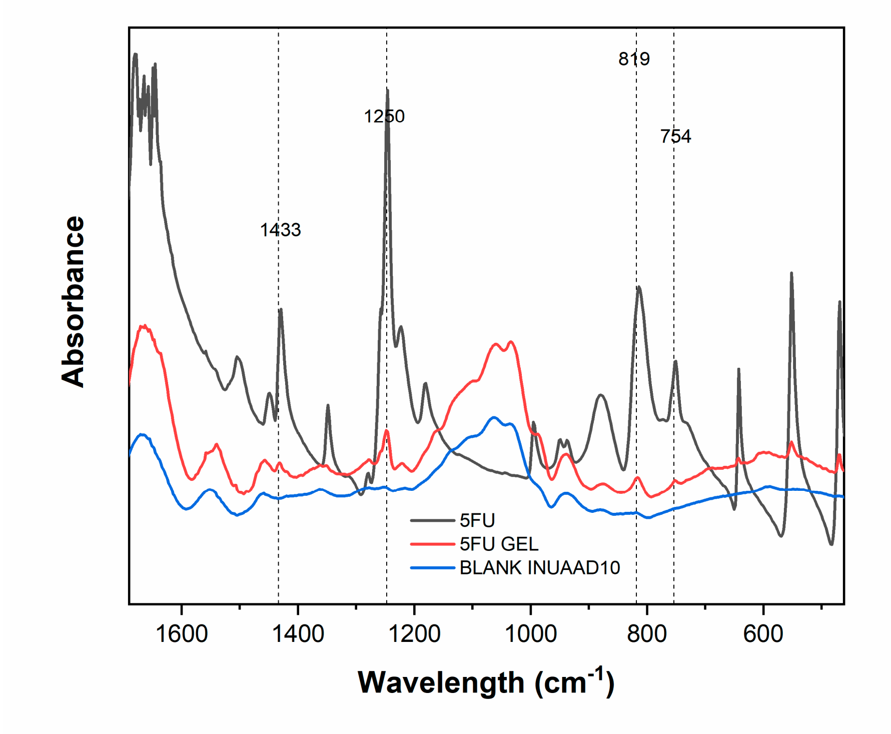

Figure S6C. FTIR spectra of pure 5FU, blank INUAAD10, and 5FU-loaded hydrogel.

Table S1. Rheological properties of INUAAD hydrogels showing the in elastic moduli (*G*′) and loss modulus (G″) at a frequency of 1 Hz.

Hydrogel	G′ (Pa)	G″(Pa)	Ratio G″/G′
**InuAAD10**	3015	290	0.096
**InuAAD5**	1928	206	0.106
**InuAAD2.5**	711.8	102	0.14

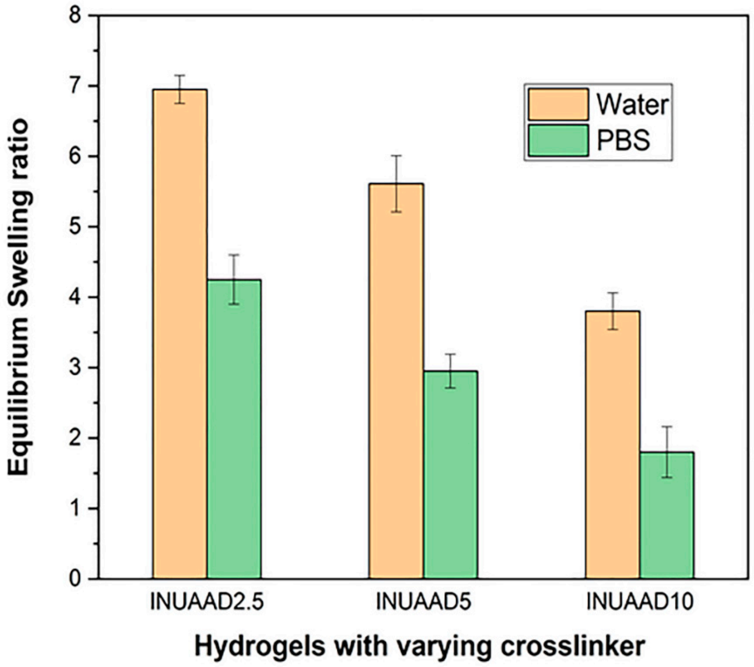

Figure S7. Equilibrium swelling of the inulin hydrogels in PBS and de-ionized water.

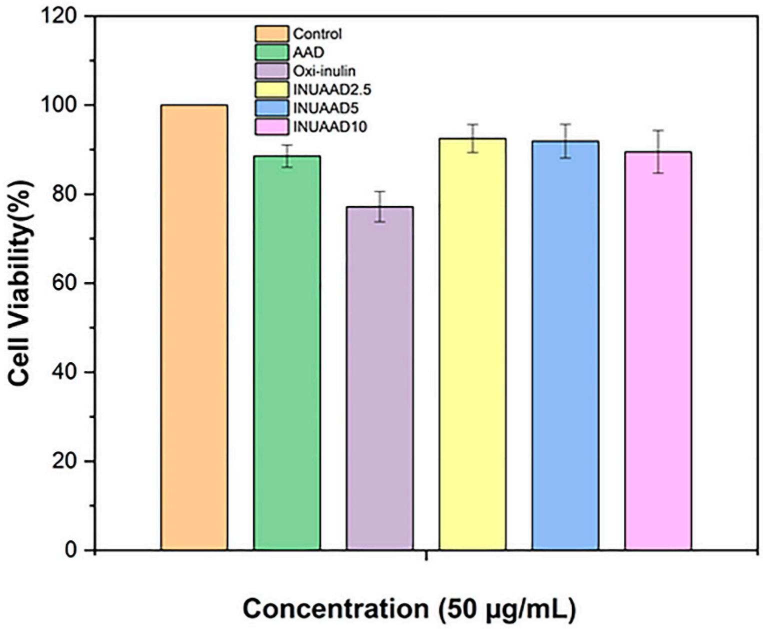

Figure S8. Cell viability of HCT116 cancer cells after treatment with 50 μg/mL of oxidized inulin, AAD, and blank hydrogels.

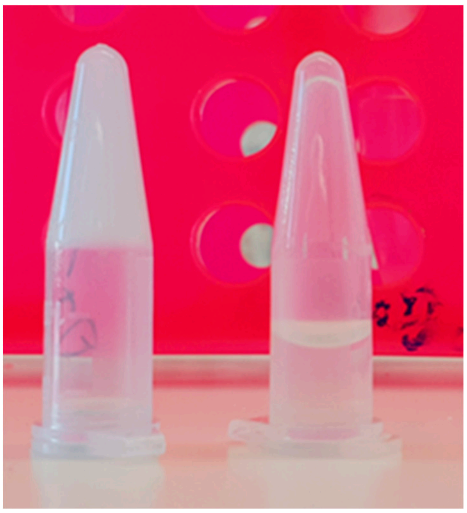

Figure S9. Crosslinking of oxidized inulin with ADD resulting in the formation of inulin hydrogel.
